# BRIDGING THE GAP BETWEEN COGNITIVE HEALTH AND FINANCIAL CAPACITY IN OLDER ADULTS: A SCOPING REVIEW

**DOI:** 10.1093/geroni/igz038.416

**Published:** 2019-11-08

**Authors:** Jessica B King McLaughlin, Ashley Taeckens, Jennifer C Greenfield, Eric Chess

**Affiliations:** University of Denver, Denver, Colorado, United States

## Abstract

Significant declines in everyday functioning are often indicators of cognitive impairment. Although researchers agree that a diminished ability to carry out a variety of instrumental activities of daily living (IADL) tasks is a harbinger of cognitive impairment (CI), studies have repeatedly found that a compromised ability to successfully complete financial management tasks may be one of the initial IADLs affected by prodromal CI. Due to the combination of compromised financial functioning and the increased prevalence of CI, older adults as group are, therefore, put at a greater risk for financial abuse and exploitation. The aims of this scoping review were two-fold: 1) to synthesize current literature on CI as it relates to financial decision-making (FDM) and 2) to analyze the measures and instruments used to assess FDM in older adults. In a review of four databases, 39 studies were identified that met inclusion criteria. These studies used 22 different instruments to measure and assess financial functioning. Across the 39 studies, consistent themes emerged relevant to factors that impacted cognitive impairment and financial functioning. These themes included participants’ education levels, premorbid financial literacy, and age. White participants were overrepresented in research samples from these studies. Future research should investigate how FDM and early signs of CI may interact in people of color. Additionally, research should be focused on how to make instrumentation more accessible and feasible for the general public to administer without extensive training. This would enable the lay population to more easily identify early signs of cognitive impairment.

